# “A bit chill, a bit silly” a qualitative study on adolescents’ views of dental clinical encounters in Norway

**DOI:** 10.1186/s12903-025-07023-w

**Published:** 2025-10-21

**Authors:** Arefe Jasbi, Anika Kurshed, Zoe Marshman

**Affiliations:** 1 Department of Design, Faculty of Architecture and Design, Norwegian University of Science and Technology (NTNU), Trondheim, Norway; 2 Changefactory, Oslo, Norway; 3 School of Clinical Dentistry, University of Sheffield, Sheffield, UK

**Keywords:** Adolescents, Children, Dental clinical encounters, Participatory design, Person-centered care

## Abstract

**Background:**

Adolescents’ perspectives on dental clinical encounters are often overlooked in oral health research. Anxiety and fear during dental visits are prevalent among adolescents, negatively impacting their oral health behaviors.

**Methods:**

This study aimed to explore adolescents’ perspectives on dental clinical encounters in Norway and identify ways to make these experiences more positive and engaging. Using participatory research methods, qualitative data were collected from 50 adolescents (aged 13–19) during a summer camp organized by Changefactory, a non-profit organization that aims to improve public services for children. Peer researchers co-developed the questions, which were delivered via digital tablets to ensure anonymous and candid responses. Thematic analysis was used to explore participants’ insights.

**Results:**

There were two main themes: (1) Comfort with the clinic’s environment and staff, emphasizing the need for informal, friendly interactions and a calming setting, and (2) Meaningful interactions, highlighting clear, honest communication, direct engagement with adolescents, and positive reinforcement through rewards or compliments.

**Conclusions:**

Incorporating adolescents’ feedback into dental practice can create more engaging and supportive clinical experiences. Participatory research methods provided deeper insights into their needs, suggesting practical interventions to improve oral health outcomes and enhance person-centered care.

**Supplementary Information:**

The online version contains supplementary material available at 10.1186/s12903-025-07023-w.

## Introduction

There has been an increasing emphasis on children’s participation in decisions that affect them following the adoption of the United Nations Convention on the Rights of the Child (UNCRC) in 1989 [1]. This includes areas such as healthcare, education, social services, and broader societal issues. Promoting children’s agency in health and healthcare aligns with theoretical models like salutogenesis. This model focuses on the positive factors that promote health and well-being rather than taking a negative disease-based approach [2].

Guidance on the teaching of paediatric dentistry and dental care for children highlights the importance of ensuring a positive experience for the patient. This experience should build a trusted relationship between the child and the dental professional with effective communication and use of appropriate techniques tailored to the needs of each child (AAPD, BSPD, IAPD guidance). However, despite this guidance, the literature suggests that visiting the dentist can cause some children and adolescents to experience fear and anxiety. Indeed, the prevalence of dental fear and anxiety has been estimated to affect 23.9% of children [3]. However, little research to date has considered children’s beliefs on how dental care can be a more positive experience for them.

Understanding adolescents’ views on dental care is essential for improving their experiences and oral health. By recognizing their perspectives, dental professionals can foster a supportive environment, reducing anxiety and fear, which often deters care. A positive experience builds trust, enhances communication, and promotes adherence to recommendations. Additionally, incorporating feedback can create more person-centered care, tailoring services to their needs and promoting shared decision-making [4, 5].

In addition to increasing children’s right to participation in healthcare, there is also a need to strengthen children’s participation in research, including oral health research [6, 7]. Within an eight-year period (2007–2015), there was a 10% shift from research *on* children to research *with* children in dental research [6, 7]. However, despite the increased use of participatory research methods with children, there remains further room for improvement [8]. Within research, children’s participation in every phase of research should be considered [9, 10]. Hart’s [11] ladder of participation describes the continuum from non-participation to full participation, where children are co-constructors in research rather than simply recipients of adult input [10]. Recognizing children’s agency, researchers can involve them in every stage of the research process, from the design phase to data collection [12], analysis, and dissemination [13].

The aim of this study was to explore adolescents’ views of dental clinical encounters in Norway, focusing particularly on how adolescents feel these experiences can be made more positive. Participatory research methods were employed, with adolescents involved throughout the research process. While the background to this paper describes general concepts related to children, the primary focus of the study is with adolescents aged 13 to 19 years.

## Method

### Ethical considerations and consent to participation

This study was part of the #Care4YongTeeth < 3 project funded by the Norwegian Research Council. The main objective of the wider project was to improve adolescents’ oral health with design interventions. The project’s research process was approved by the Norwegian Agency for Share Services in Education and Research (Sikt). During Changefactory’s summer camp, all participants gave written informed consent after receiving clear information about the study’s aims, methods, and their rights, including the option to withdraw at any point. For those under 16 years of age, consent was obtained from both the child and their parent or legal guardian. Participants aged 16 and above, provided consent independently. All collected data were anonymized, and no personal identifiers were recorded.

### Research design and participants

This exploratory study is based on written qualitative data from adolescents attending a summer camp in 2022, arranged by Changefactory. Changefactory is a non-profit organization created to collect experience and advice from children and young people and give them an opportunity to improve public services [14]. Changefactory employs various approaches that are central to the organization’s efforts in building long-lasting, trust-based relationships with children and young people. At these annual summer camps, adolescents aged 13 to 19 were invited by Changefactory to take part. The camps are free events for young people who wish to provide advice on various public services they have been in contact with.

Using participatory methods, academic researchers collaborated with peer researchers from Changefactory who were involved in data collection, analysis, and reporting. The open-ended questions were co-created at a brainstorming session and a round of revising by both peer and academic researchers (A.J. and Z.M.) and covered adolescents’ views of clinical encounters with dental professionals during visits to dental clinics, as well as how these experiences could be enhanced positively (Appendix A). The questions were initially developed in English and subsequently translated into Norwegian by native Norwegian peer researchers who are also fluent in English and familiar with the cultural context to meet the adolescents’ language. A.J. back-translated the question to verify that the translated questionnaire preserved the original meaning.

All participants consented to participate in the camp, where they provided advice on various services, including healthcare services. Participants under 18 years of age obtained consent from their guardians to participate. All participants at the camp were invited to partake in this study. The participants consisted of a convenience sample of students from middle and high schools throughout Norway. Fifty participants aged 13 to 19, including about 75% females and 25% males. The camps lasted for five days each. The survey was conducted on the third day. Data collection was conducted as a group activity with groups of five or six participants of mixed genders. The final sample size of 50 participants was determined by the number of adolescents attending the summer camp and the number required to reach data adequacy, considering the nature of the participants, the questions asked of the groups, and the analysis conducted.

Each participant received a tablet with an anonymous questionnaire from the peer researcher assigned to their group. The peer researcher first explained the task, ensuring the participants understood the information. Prior to the survey, all of the young people at the camp were informed about how their responses would be used, that the survey was anonymous, and who would receive the anonymized answers. They were also informed that participation and answering the questions were voluntary. The participants were told that their age and gender would be recorded, but this data would not be shared. The peer researchers were present and available for questions and help if needed. The participants had approximately 30 min to answer and submit their responses anonymously. At the end of the 30 min, the peer researchers ensured everyone submitted their answers and made the tablets ready for the next group.

### Data analysis

A thematic analysis was conducted following the structured approach outlined by Braun and Clarke [15]. The process began with an initial familiarization with the data, where researchers read through all responses multiple times.

An inductive, data-driven approach was used to generate initial themes. Coding was performed manually, with the peer researchers collaboratively identifying the first set of themes and organizing the responses. Academic researchers (A.J. and Z.M.) then reviewed and refined these themes, ensuring clarity and coherence before discussing them with peer researchers to finalize the categorization.

To ensure the themes accurately represented the data, responses assigned to each theme were reviewed and, in some cases, rearranged to better fit emerging patterns. During the refinement process, some themes were merged, split, or removed as necessary. No disagreements arose during the thematic analysis. The identification and refinement of themes followed a consensus-based approach, with peer and academic researchers working collaboratively throughout the process.

The analysis was conducted manually, following a structured and iterative process to systematically identify key themes.

## Results

**Fig. 1:**
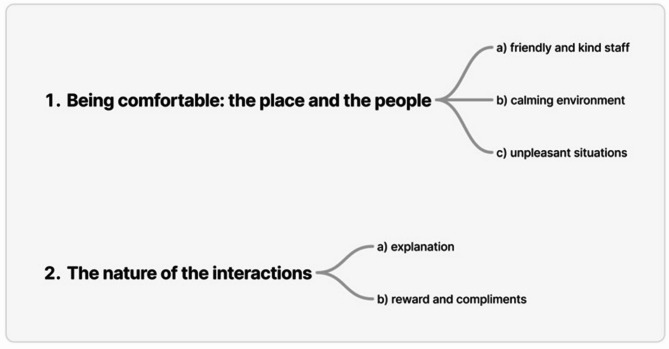
Overview of themes and sub-themes

The findings revealed the views of adolescents about encounters within the dental setting. The analysis identified two main themes (Fig. 1). The first theme was being comfortable with the people and the place, which included sub-themes: (a) friendly and kind staff, (b) calming environment, and (c) unpleasant situations. The second theme was the nature of interactions, which includes sub-themes of (a) explanation and (b) rewards and compliments.

### Being comfortable: the place and the people

The first theme described how the people in the clinic and the physical environment of the clinic were perceived to create a positive experience or, indeed, what made the experience tend to be perceived more negatively. Adolescents expressed a preference for informal and friendly staff within a calming environment.

#### Friendly and kind staff

Adolescents reported various attitudes and attributes they expected from the staff in dental clinics, extending beyond the dentist to include assistants and receptionists. They shared positive and negative experiences, highlighting the importance of a comfortable and low-pressure environment, including the use of humor.

*“Had the same [dentist] since I was a tiny [child]. She’s been like… when I come in ‘what was the name? No*,* it’s you again!…’.”*

*“Be funny… before*,* during*,* and after the dental appointments.”*

Creating a casual and friendly atmosphere was also emphasized. Adolescents appreciated it when staff took a relaxed approach characterized by their general demeanor and clothes.

*“down to earth*,* not over the top*,* a bit chill*,* a bit silly*,*”*

*“I like the patience… when they’re not rushing.”*

*“…singing along to the radio during treatment.”*

*“…he wears colored shirts. Color every single time… Looks like he’s on vacation at work.”*

They highlighted the stress-relieving effect of kind conversations and preferred these interactions to start in the treatment room to avoid embarrassment in the waiting area.

*“Talk a little when you enter. May feel a little scared and anxious.”*

Building connection was another critical aspect, typically expressed as the dental professional ‘getting to know’ the patient with suggested topics of conversation as: future aspirations, school, hobbies, and storytelling.

*“Get to know each other because then we will be equal… because then it will be a little more pleasant to be in the stupid room.”*

However, negative experiences were characterized as involving unfriendly staff, impatience, anger, and a lack of empathy. Negative interactions, such as being shouted at, were particularly distressing:

*“…got mad at me when I didn’t like smells. He was pissed off. He screamed at me…”*,

*“They yell at you if they see a mistake. Feels very scary. Won’t be coming back to the dentist unless I get one that isn’t like that…"*.

These views highlight the importance of having friendly and kind staff who can create a welcoming and stress-free atmosphere. However, it is equally important to focus on the physical environment, ensuring it is calming and engaging.

#### Calming environment

Adolescents reported several suggestions for making the environment more comfortable at the clinic, both in the treatment and waiting rooms. Some expressed a preference for maintaining a connection with the ‘outside world’ by having a window to look through:

*“more life inside the room*,* [it is] scary sitting there for ages.” “*,* the chair facing outwards*,* you can see things happening outside*,*” “The view of a center*,* can see what people are doing.”*

Distractions like films, posters, and music were also welcomed to help pass the time and reduce anxiety:

*“Film on the ceiling. It is a piece of advice*,* children who are afraid or have poor concentration*,* have to sit still for a long time*,* cannot sit and look at a white ceiling.”*

“In *** there is a dentist’s chair,* then there is a really busy poster*,* been there several times*,* never managed to look at the whole poster*,* stare at it for hours…"*.

Music, in particular, was highlighted as a calming factor:

*“Having music*,* something catchy for the child*,* not radio. Not boring music*,* maybe let the child decide for himself.”*

These suggestions collectively point to the importance of a soothing and engaging environment to help adolescents feel more at ease during their visits to the dental clinic.

#### Unpleasant situations

Despite efforts to create a positive environment, some adolescents described situations that were unpleasant and uncomfortable:

*“When they are completely on top of you*,* they get their tits [i.e. breast] in your face. [It] is uncomfortable.”*

They expressed discomfort with specific procedures, stating sarcastically,

“It’s reeeally f***ing nice - jelly lump in the throat.”

There were also concerns about when dentists do not ask patients before they start touching their mouths:

*“[they] don’t ask you to open your mouth…they open your mouth with their hands… Unpleasant…NO just NO.”*

The use of metal instruments was particularly unsettling:

*“…in my mouth with such metal things.”*

In summary, this first theme captured what made adolescents comfortable within a dental setting both in terms of the attributes of the staff and the physical environment.

### The nature of the interactions

The second theme focused on the interactions with two sub-themes: explanations and how rewards and compliments can make the interaction more positive. Adolescents desired meaningful interactions with their dentists, emphasizing the importance of clear communication to promote understanding, and the need for personalization. They wanted to establish a direct relationship with the dentist, focusing on themselves rather than involving their parents. The explanations given by the dentist should be interesting, non-judgmental, and free of jargon. Adolescents needed to feel they could ask questions if they did not understand something.

#### Explanations

They valued learning about ways to improve their oral health behaviors from various sources, including schools, health centers, youth centers, dental clinics, and social media.

*“Social Media. That’s where we are. Where we spend 90% of our time…"*.

There were suggestions advocating for widespread dental education, stating,

*“It should be a bit everywhere*,* kindergarten*,* TV*,* school*,* home*,* etc.”*

They even proposed having a dentist give presentations at school instead of a teacher, as it would feel more credible:

*“Maybe if a dentist comes and gives a presentation at school. Not the teacher. Better with a dentist who has knowledge. If the maths teacher says something about teeth*,* it gets a little weird.”*

In the clinical setting, adolescents appreciated when the dentist provided them with a plan and explained procedures during the treatment. Indeed, some stated a dislike for silences as they made them feel uninformed and anxious:

*“… Make a plan for you so you know what happens next*,* so you don’t have to come and then not know. Children and young people are afraid of the dentist. It was very nice to have a plan. …” “The dentist can explain while doing it. Talk a little about the teeth along the way.”*

*“Hate when they are completely silent and don’t say a damn thing. You don’t know what’s going on*,* what they’re doing. You feel like a lab rat being experimented on.”*

Adolescents stressed that honesty is crucial and that perceived dishonesty, particularly about the potential for pain, can result in a loss of trust:

*“… don’t like that they lie about it hurting. You lose confidence…knowing that the dentist is not honest. If they are going to inject an anesthetic*,* say that it may sting or hurt. …"*.

They also disliked being blamed or shamed for their dental issues:

*“Don’t say it’s my fault I don’t brush*,* and don’t mention that the dentist is damn expensive.”*

“no need to say that the teeth are f***** and that you make a lot of mistakes. Can say that you should brush more,* not that the teeth will rot.”*

Interactions with the staff could also be problematic when participants felt they could not speak freely, when they felt initiating a conversation was awkward or when they were not listened to.

Direct communication with the adolescent rather than their parents is preferred:

*“…Talk to the child rather than the parents…” and*.

*“Do not talk to the parents first without the child. The child is left in the hallway and feels insecure…"*.

Adolescents believed it was important for them to receive information firsthand about their dental health:

*“Get information directly. Coming straight to us. Do not talk to the assistant. Talk to us honestly so we know too.”*

The tone and language used by the dentist significantly impact the adolescents’ comfort. They appreciated a friendly, non-judgmental tone:

*“Mine is really cool. Says in a nice*,* non-judgmental way.”*

They disliked monotonous or technical language:

*“… they have to be careful not to be so monotonous in their voice. … Don’t use technical terms*,* don’t understand a damn thing*,*”*

*“Don’t talk technical language/weird because I haven’t learned those words. I haven’t been to school for as many years as them.”*

#### Rewards and compliments

Adolescents expressed a preference for a positive outcome of the visit, either in terms of a physical reward or a compliment for making progress, viewing this as a positive reinforcement, which was motivational and made dental visits more enjoyable:

*“I feel it’s a bit silly that they say I’m too big for a prize*,* I want a prize every time.”*

*“Yes*,* must get prizes regardless of age.”*

*“If they give compliments*,* don’t just look at the negatives; they look at the positives a bit too.”*

*“When the dentist says remember to brush your teeth*,* I know you can do it. HAS HAPPENED.”*

*“My last one did it*,* she just did such a good job*,* she gave me a hug*,* took my hand*,* things like that that make me a little more motivated to go to [the clinic].”*

*“When they gave me compliments that I had nice teeth*,* I was given hope that I have good dental health.”*

In summary, the adolescents emphasized the importance of meaningful and positive interactions with their dentists, highlighting the need for clear communication, honesty, and personalization. They valued explanations and rewards as tools for motivation, appreciating a friendly and non-judgmental approach that includes direct communication and positive reinforcement.

## Discussion

The study explored adolescents’ views about clinical encounters in Norway. Overall, the findings revealed what made adolescents feel more comfortable in their encounters, their preferences for the physical environment, and staff interactions with an emphasis on adolescents’ beliefs of the positive aspects of these encounters. Giving adolescents agency as co-researchers in this study allowed a fuller understanding of their views that adults may otherwise have overlooked.

Adolescents’ feedback about dental experiences was shaped by a combination of the clinic environment and the nature of staff interactions. They emphasized the importance of informality, humor, and staff who fostered a relaxed and welcoming atmosphere. Indeed, paediatric dentistry guidance suggests the use of humor and friendly, non-verbal communication strategies as being considered effective in reducing anxiety and establishing trust [16, 17]. While positive experiences can help alleviate anxiety, negative experiences, such as being shouted at or dismissed, can be particularly damaging, often leading to reluctance to return (AAPD, 2024).

To improve their overall satisfaction with clinical encounters, adolescents express a desire for more involvement in discussions about treatment decisions through direct communication with dental professionals rather than through their parents [16, 18]. Such a desire for shared decision-making fits well with paediatric dentistry guidance and the patient-driven environment of health services in Norway [19–21]. This study suggests clear, jargon-free explanations and candid discussions are expected, giving adolescents a greater sense of control over their healthcare experience, although this may not be the expectation in other countries with different approaches to children’s agency in healthcare predominate.

Rewards, such as small prizes or compliments, were perceived positively as motivational for adolescents in improving their oral health behaviors, as identified in this study and our previous work [21]. Adolescents appreciated their positive oral health behaviours to be acknowledged, and the positive reinforcement associated with rewards was viewed as a powerful tool for motivation [22, 23]. Additionally, the physical environment of the clinic, for example, with calming music and visuals to distract, helps minimize anxiety and increase comfort, making the facility feel less clinical and more approachable for adolescents [24, 25].

Overall, the feedback aligns with broad research evidence supporting a holistic approach to adolescent dental care. It emphasizes that a supportive and positive environment [2], both in the physical space and in staff interactions, contributes to making the experience more engaging and less disease-focused.

### Study design

The level of children’s participation in this study reached the highest ‘rungs’ of Hart’s ladder of participation: child-initiated and directed, shared decisions with adults, where both children and adults collaborate, respecting each other’s perspectives and contributions [11]. These high degrees of participation advocate for children’s agency, ownership, and the recognition that children can contribute valuable insights and lead initiatives [10, 11]. Although peer researchers have also been involved similarly in other studies [26], this level of participation is rare in child oral health research [8].

The Changefactory’s approach centers on making a safe and trusting environment with adolescents before initiating the data collection process. The method of data collection (via tablet computers) and questions posed were co-created by the peer researchers to ensure their appropriateness and to make them comprehensible to a wide range of cognitive and language capacities [8]. These approaches were chosen to help adolescents feel more comfortable participating and to encourage more authentic responses free from the power dynamics with adult researchers [27]. It appeared that participants’ answers were marked by greater honesty, use of casual language, occasional swearing, and sarcasm. This apparently contrasts with interviews conducted by adults, where responses may be moderated, with participants potentially tailoring their answers to align with perceived expectations.

It is particularly important to gain a holistic understanding of adolescents’ beliefs when these views are captured to feed into the co-design of new interventions or service re-design.

### Strengths and limitations

The strengths of this study were the inclusion of peer researchers to make the study relevant and the positive stance taken, which, rather than focusing on dental fear and anxiety, sought to emphasize the positive aspects of dental encounters.

However, one limitation of this study was that a convenience rather than a purposive sample of participants was recruited. Within qualitative research, typically a purposive sample is recruited to ensure a diverse range of views are obtained, with data collected about participants’ socio-demographic characteristics to describe that diversity. However, as the consent process relied on participants remaining anonymous, it was not possible to provide that description of the sample (e.g., age or gender), although the data does suggest a range of views was observed.

The generalizability of the findings is also a limitation, given the sampling technique and the predominance of female participants. While the data were collected from adolescents experiencing the Norwegian dental services, which may partially reflect the interactions between patients and clinicians in that system, the findings do have wider implications for pediatric dentistry services and indeed the teaching and training of dental professionals who care for children.

While we focused on adolescents’ perspectives, future research should explore dentists’ viewpoints to improve the relevance of these findings for both patients and providers.

## Supplementary Information

Supplementary Material 1.

## Data Availability

Anonymized transcripts and notes that support the findings of this study are available from the corresponding author, upon reasonable request.
