# Supplementary material for: “A bit chill, a bit silly” a qualitative study on adolescents’ views of dental clinical encounters in Norway
Source: BMC Oral Health. 2025 Oct 21;25:1644. doi: 10.1186/s12903-025-07023-w (PMC12538737; doi:10.1186/s12903-025-07023-w)
Supplement: Supplementary file 1 — Supplementary Material 1. [file 12903_2025_7023_MOESM1_ESM.docx]

1. Is there anything you like about your dentist/dental clinic?

2. Is there anything you don't like about your dentist/dental clinic?

3. Do you have an example of something that has given you hope when you have been to the dentist?

4. When children are at the dentist, is there anything the dentist can do that can make children happy?

5. Is there anything the dentist should not say or do that makes it unsafe?

6. How can the dentist/dental health service help young people to want to take care of their teeth?

7. Where do young people want to learn about taking care of their teeth? From whom? In what way?
